# HTK vs. HTK-N for Coronary Endothelial Protection during Hypothermic, Oxygenated Perfusion of Hearts Donated after Circulatory Death

**DOI:** 10.3390/ijms25042262

**Published:** 2024-02-13

**Authors:** Lars Saemann, Kristin Wächter, Nitin Gharpure, Sabine Pohl, Fabio Hoorn, Sevil Korkmaz-Icöz, Matthias Karck, Gábor Veres, Andreas Simm, Gábor Szabó

**Affiliations:** 1 Department of Cardiac Surgery, University Hospital Halle (Saale), University of Halle, 06120 Halle (Saale), Germany; 2 Department of Cardiac Surgery, University Hospital Heidelberg, 69120 Heidelberg, Germany

**Keywords:** heart transplantation, donation after circulatory death, machine perfusion, organ preservation, coronary endothelium, endothelium, Bretschneider, Custodiol, Custodiol-N

## Abstract

Protection of the coronary arteries during donor heart maintenance is pivotal to improve results and prevent the development of coronary allograft vasculopathy. The effect of hypothermic, oxygenated perfusion (HOP) with the traditional HTK and the novel HTK-N solution on the coronary microvasculature of donation-after-circulatory-death (DCD) hearts is known. However, the effect on the coronary macrovasculature is unknown. Thus, we maintained porcine DCD hearts by HOP with HTK or HTK-N for 4 h, followed by transplantation-equivalent reperfusion with blood for 2 h. Then, we removed the left anterior descending coronary artery (LAD) and compared the endothelial-dependent and -independent vasomotor function of both groups using bradykinin and sodium-nitroprusside (SNP). We also determined the transcriptome of LAD samples using microarrays. The endothelial-dependent relaxation was significantly better after HOP with HTK-N. The endothelial-independent relaxation was comparable between both groups. In total, 257 genes were expressed higher, and 668 genes were expressed lower in the HTK-N group. Upregulated genes/pathways were involved in endothelial and vascular smooth muscle cell preservation and heart development. Downregulated genes were related to ischemia/reperfusion injury, oxidative stress, mitochondrion organization, and immune reaction. The novel HTK-N solution preserves the endothelial function of DCD heart coronary arteries more effectively than traditional HTK.

## 1. Introduction

According to recent advances in the technologization of donor organ preservation, new devices were developed to maintain donor hearts by ex-vivo machine perfusion either in a beating state or in a resting state. Beating heart maintenance is performed using normothermic, oxygenated blood perfusion. Resting heart preservation is performed using static cold storage or hypothermic, oxygenated perfusion (HOP), either fully crystalloid or with added erythrocytes [1,2]. Ex-vivo machine perfusion allows, first, postconditioning of the heart after procurement, especially in the case of donation after circulatory death (DCD), second, protection during transportation, and third, preconditioning before reperfusion in the recipient. Ex-vivo machine perfusion with normothermic blood enabled the development of DCD heart transplantation programs. Nevertheless, different considerations have been published on whether or not it is necessary to observe the heart beating during transportation or if a non-beating transport, including cold storage, is sufficient by the group of Large and colleagues [3].

During reperfusion after transplantation, multiple mechanisms can deteriorate graft function. However, most attention is being paid to alleviating the detrimental effects on the myocardium, which addresses most notably primary graft dysfunction. Nevertheless, the coronary vasculature is pivotal for long-term graft survival. Besides immunological effects, ischemia/reperfusion (I/R) injury of the coronary artery endothelium is a proven trigger of coronary allograft vasculopathy (CAV), which limits long-term survival after cardiac transplantation [4].

In the coronary micro- and macrovasculature, I/R injury, oxidative stress, acute immune responses, and cell survival or cell death pathways must be considered to maintain a functionally intact endothelium and smooth muscle cells and perhaps initiate or promote tissue regeneration. In DCD hearts, the warm ischemia before procurement is an additional ischemic noxa of the coronary endothelium. Our novel histidine-tryptophane-ketoglutarate-N (HTK-N) preservation solution was developed with the special purpose of preserving the coronary endothelium as well. It was already shown that HTK-N reduces myocardial I/R injury after heterotopic heart transplantation in rats [5,6]. We have also compared the microvascular endothelial effects of HOP with HTK and HTK-N, including an analysis of preselected genes in a porcine model of DCD [7]. However, coronary artery disease also affects the macrovasculature, which is a common complication after orthotopic heart transplantation [8]. Thus, we investigated the effect of HOP of DCD hearts with the HTK-N solution on the endothelial function and transcriptome of the left anterior coronary arteries.

## 2. Results

### 2.1. Vasomotor Function

**Figure 1:**
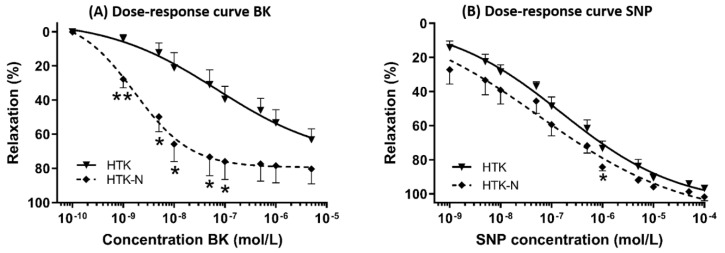
Dose-response curves. (**A**) Dose-response curve for endothelial-dependent vasorelaxation to bradykinin. (**B**) Dose-response curve for endothelial-independent vasorelaxation to sodium-nitroprusside. BK: bradykinin. HTK: histidine-tryptophane-ketoglutarate. HTK-N: histidine-tryptophane-ketoglutarate-N. SNP: sodium nitroprusside. N = 8 per group. * *p* < 0.05 vs. HTK. ** *p* < 0.001 vs. HTK.

**Figure 2:**
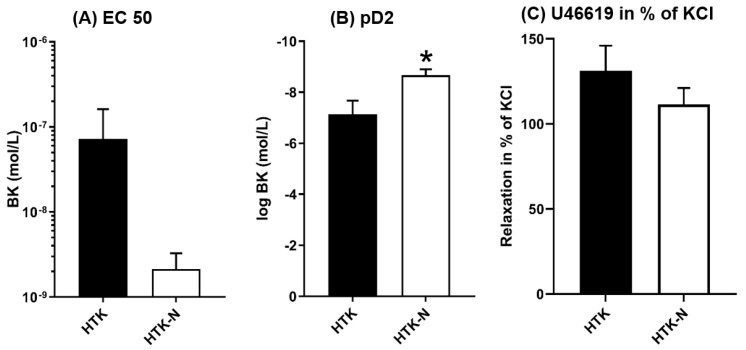
Characteristic functional parameters. BK: bradykinin. EC: effective concentration. HTK: histidine-tryptophane-ketoglutarate. HTK-N: histidine-tryptophane-ketoglutarate-N. KCL: potassium chloride. pD2: logEC50. SNP: sodium nitroprusside. N = 8 per group. * *p* < 0.05 vs HTK.

The endothelial-dependent relaxation of the LAD to bradykinin was significantly better after perfusion with HTK-N (Figure 1A). The endothelial-independent relaxation to SNP was comparable between both groups (Figure 1B). The EC50 and pD2 of bradykinin were lower and thus superior in the HTK-N group (Figure 2A,B). The contraction to the thromboxane A_2_ agonist U46619, normalized to the contraction to potassium chloride, was lower after HTK-N perfusion (Figure 2C).

### 2.2. Transcriptome

**Figure 3:**
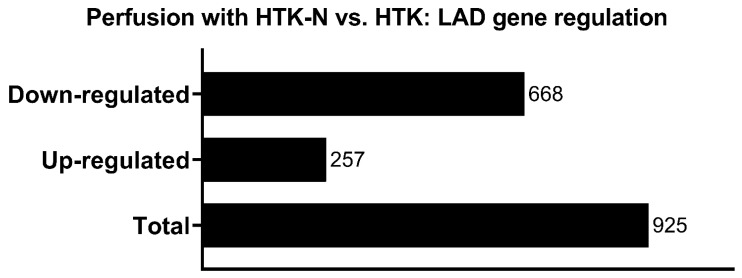
Regulated genes in HTK-N vs. HTK.

**Figure 4:**
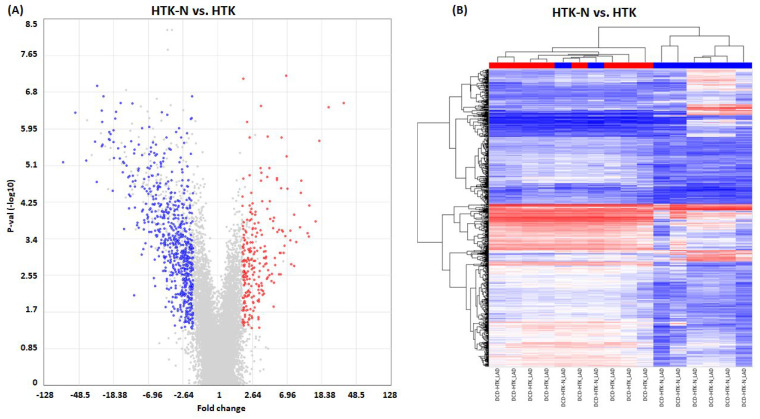
Gene regulation. (**A**) Scatter plot. (**B**) Heat map. HTK: histidine-tryptophane-ketoglutarate. HTK-N: histidine-tryptophane-ketoglutarate-N. KCL: potassium chloride. N = 8 per group. Blue: downregulated transcripts. Red: upregulated transcripts.

In total, 925 genes were significantly regulated in the LAD of the HTK-N group compared to the HTK group (Figure 3). Of those, 257 genes were expressed higher, and 668 genes were expressed lower (Figure 4). The top 20 genes that were most upregulated or downregulated are shown in Table 1 and Table 2.

In the LAD of the HTK-N hearts, several genes that are involved in endothelial cell functionality, such as ACTB or ACTG1 and RGS5, preservation of endothelial cell junctions, such as SMAD6, as well as vascular smooth muscle cells preservation, such as TMSB4X, were upregulated. In addition, transcripts involved in attenuating oxidative stress, such as CEBPD and ATN1, were also upregulated. Other overexpressed transcripts are involved in regulating and protecting from I/R injury, such as ZFP36L2 and MIR505. Transcripts involved in survival and regeneration after ischemia, such as PIM1 and FOXC2, were also upregulated. Elevated transcripts related to immune response were IER3, ZFP36, and CCL2. Further upregulated transcripts were GJA1, SNAI2, PNRC1, RNF149, BTG2, COL3A1 and SLC39A14.

Transcripts expressed lower in the LAD of HTK-N hearts compared to HTK hearts are associated with I/R, such as NDUFS4, and oxidative stress, such as MRPS18C and NDUFC2. Other downregulated genes, such as NDUFB1 and NDUFB2, ROMO1, and COX7A2, are relevant for mitochondrial function. Some genes related to immunity and immune reaction, such as SNRPG, were also expressed lower in the LAD of HTK-N hearts. Other downregulated genes, such as NDUFA1 and NDUFB8, are related to coronary artery disease. Further downregulated genes were RNF113A, HSPE1, CHCHD7, MGST2, USMG5, TBCA, and IGIP.

**Figure 5:**
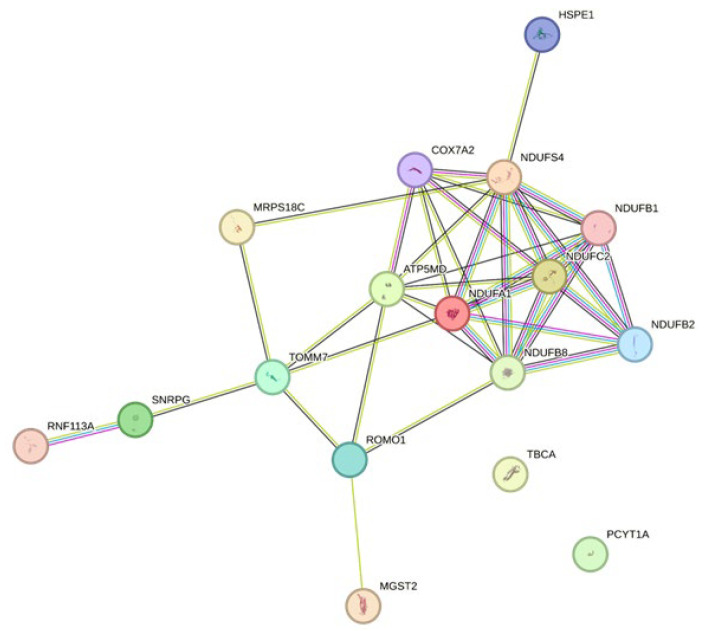
Network analysis of the top 20 downregulated genes of HTK-N vs. HTK. The network was built utilizing the Gene String online tool (STRING: functional protein association networks (string-db.org; assessed date: 18 January 2024). HTK: histidine-tryptophane-ketoglutarate. HTK-N: histidine-tryptophane-ketoglutarate-N.

The network analysis revealed a dense network for the downregulated genes (Figure 5). The enriched pathways within this network were related to the electron transport chain and mitochondria. The top 20 upregulated genes did not show a network with interacting genes. Thus, this network is shown in the Supplementary Materials (Figure S1).

### 2.3. Pathways

**Figure 6:**
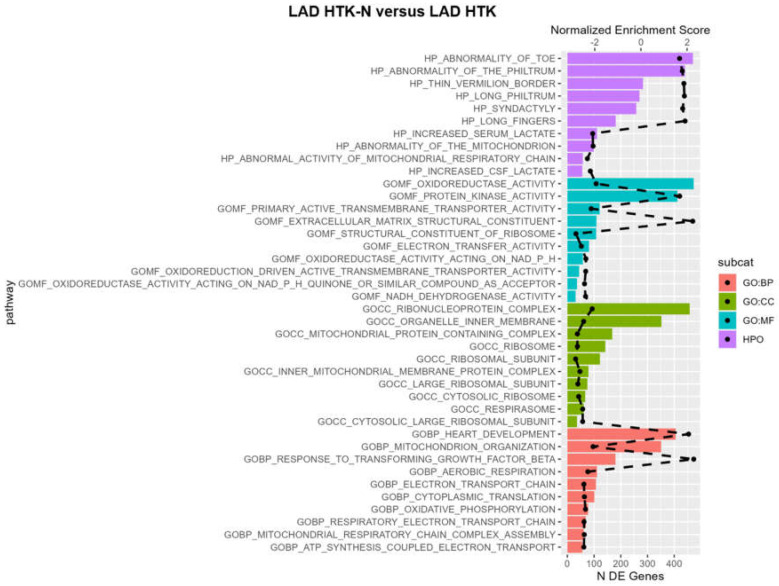
Pathway analysis. BP: biological process. DE: differentially expressed. CC: cellular component. GO: gene ontology. HTK: histidine-tryptophane-ketoglutarate. HTK-N: histidine-tryptophane-ketoglutarate-N. HPO: human phenotype ontology. MF: molecular function. The dashed black line represents the normalized enrichment score. All plotted pathways are *p* < 0.05. N = 8 per group.

The pathway analysis revealed that in the LAD of DCD hearts, maintained by perfusion with HTK-N, enriched pathways were mainly associated with oxidoreductase (Figure 6). Oxidoreductase activity-related functions were upregulated in the HTK-N group compared to the HTK group. Oxidoreduction-driven transmembrane transporter activity and NADH dehydrogenase activity were downregulated. Pathways related to protein kinase activity and extracellular matrix structural constituents were upregulated. Regarding cellular components, the top ten pathways were all downregulated, such as those associated with the organelle inner membrane, ribonucleoprotein complex, ribosome, ribosome subunit, and cytosolic ribosome. Regarding biological processes, pathways related to mitochondrion organization, aerobic respiration, and oxidative phosphorylation were underrepresented in the HTK-N group. Pathways associated with heart development were upregulated.

## 3. Discussion

In the present study, we compared the effect of HOP of porcine DCD hearts with the traditional HTK and the novel HTK-N solution, also known under the commercial name Custodiol and Custodiol-N, on the endothelial function and transcriptome of the LAD.

### 3.1. Functional Effects

The higher endothelial-dependent relaxation in the HTK-N group reveals superior endothelial preservation of the LAD of DCD hearts by HOP using the novel HTK-N compared to traditional HTK. The similar endothelial-independent relaxation of both groups confirms that the bradykinin-associated effects are indeed based on endothelial function and not on smooth muscle cell function defects. The lower pD2 and EC50 also suggest a better endothelial function in the HTK-N group. According to the reconditioned microvascular function that we already showed in a previous publication, HOP with the novel HTK-N solution also improves the macrovascular function of DCD hearts compared to HOP with traditional HTK [7]. The last study revealed a better myocardial microcirculation during reperfusion with blood in hearts maintained by HOP with HTK-N compared to those maintained by HOP with HTK. Different from the previous study, in the present study, we applied pharmacological agents to investigate the endothelial-dependent and independent vasomotor function specifically instead of the effect of different coronary perfusion pressures that allow investigation of coronary autoregulation.

### 3.2. Transcriptome Effects

#### 3.2.1. Endothelial Cell Functionality

The upregulated transcripts ACTB and ACTG1 are important for endothelial cell functionality and could explain the high endothelial-dependent relaxation in the HTK-N group [9]. SMAD6 transduces the endothelial cell flow responses required for blood vessel homeostasis [10]. Additionally, a loss of SMAD6 leads to disrupted endothelial cell junctions [11]. Thus, the decreased expression of SMAD6 in the LAD of the HTK group might have contributed to the impaired functional characteristics in this group. It is also known that the downregulation of RGS5 impairs endothelial cell function and contributes to coronary artery disease. It might also have contributed to the decreased endothelial-dependent vasomotor function in the HTK group [12]. TMSB4X preserves the vascular smooth muscle phenotype in atherosclerosis and leads to vascular smooth muscle cell development and vessel wall stability, presumably explaining the superior results [13].

#### 3.2.2. Ischemia/Reperfusion and Oxidative Stress

I/R injury deteriorates tissue and organ function by various pathomechanisms. Consequently, tremendous efforts are being made to reduce I/R. Meanwhile, many genes that contribute to I/R injury have been identified. Of those, some were also significantly regulated in the present study. ZFP36L2 regulates myocardial I/R injury [14]. MIR505 might attenuate myocardial ischemia/reperfusion injury [15]. Both publications are related to the myocardial effect. However, similar effects might occur in vascular I/R and could explain the functional differences between both groups. In addition, Zhang et al. showed that a heart-specific knockout of NDUFS4 ameliorates I/R injury [16]. Thus, the downregulation of NDUFS4 could also have been the reason for the superior vasomotor function in the HTK-N group.

Oxidative stress is a major contributor to the manifestation of I/R injury. ANT1, expressed higher in the HTK-N group, attenuates reactive oxygen species production and stabilizes the mitochondrial membrane potential by blocking the opening of the mitochondrial permeability transition pore [17]. Thus, ANT1 is important in anti-oxidative cell-protective processes [18]. CEBPD reduces inflammation and oxidative stress in cardiomyocytes in cell culture and presumably also in the LAD of the HTK-N hearts [19]. MRPS18C seems to be a marker for oxidative stress. Thus, its low expression reflects a low level of oxidative stress in the LAD of HTK-N hearts [20]. NDUFC2 is involved in providing the antioxidant capacity of the endothelium during I/R and might have been downregulated because no elevated antioxidant mechanisms were needed due to the seemingly reduced oxidative stress [21]. I/R-related effects, as well as oxidative stress that seems to be reduced in the LAD of the HTK-N group, are in line with the results from a previous publication, which showed reduced ischemic injury and a lower level of oxidative stress in the myocardium, confirmed by immunohistochemical staining [22]. However, based on transcriptomics, the current study identified all significantly regulated genes related to I/R and oxidative stress in the LAD compared to previous studies. Nevertheless, the potential effects on the protein level are still unknown.

#### 3.2.3. Immune Reaction and Survival

ZFP36 is typically expressed in vascular endothelial cells or macrophage foam cells, and it inhibits the expression of proinflammatory mRNA transcripts and consequently has anti-inflammatory effects, which might also have improved relaxative properties in the HTK-N group [23]. In murine cardiac allografts, IER3 was associated with activated B cells [24]. However, it is also part of the NUPR1/RELB/IER3 survival pathway [25]. The upregulated PIM1 kinase in the HTK-N group promotes angiogenesis through phosphorylation of endothelial nitric oxide synthase [26] and improves survival of cells after ischemia [27]. SNRPG is responsible for T-cell activation in heart failure patients [28]. Thus, it may contribute to immune activation when upregulated in the LAD of DCD hearts after HOP with traditional HTK.

#### 3.2.4. Regeneration

Following a harmful noxa, regenerating cells, tissues, and organs is highly important. Thus, the upregulation of FOXC2, which is involved in developing and functioning endothelial progenitor cells that contribute to vascular regeneration in coronary artery disease, might have promoted vascular regeneration in the DCD allograft of the HTK-N group [29].

#### 3.2.5. Mitochondrial Relevance

Mitochondria are important subcellular components involved in the consequences of I/R injury. Mitochondrial dysfunction contributes to cell dysfunction and dysfunctional organs. NDUFB1 and NDUFB2 are associated with altered cardiac energetics and mitochondrial dysfunction in patients with hypertrophic cardiomyopathy, and ROMO1 is an essential redox-dependent regulator of mitochondrial dynamics [30,31]. Consequently, the mitochondria in the LAD of the HTK group could have been functionally impaired.

#### 3.2.6. Coronary Artery Disease Associations

NDUFB8 and NDUFA1 are known from patients with coronary artery disease (CAD). NDUFB8 is increased in patients with (stable) CAD and was shown to be decreased in the blood of patients with CAD after completion of a rehabilitation program [32,33]. Thus, the lower expression in the HTK-N group alludes to a more vital coronary artery status.

### 3.3. Associations with Graft Dysfunction

Halloran and colleagues recently published a microarray-based analysis of gene expression from patients with graft dysfunction or graft loss within three years after biopsy [34]. Of note, biopsies were taken from endomyocardial tissue and not LAD. Nevertheless, we compared the top 20 regulated genes associated with graft dysfunction and those associated with graft survival with the top 20 up and downregulated transcripts of our work. FBLN1 (FC = 2.08; *p* < 0.05) and DENND2A (FC = 1.4; *p* < 0.05) were expressed lower in the group with graft dysfunction. Both were also downregulated in the HTK group. LUM (FC = 1.93; *p* = 0.7405) was associated with graft survival and was increased in the HTK-N group. A comparison of the gene expression of both groups from our work with the transcriptome of LAD samples from patients with and without CAV would be very expedient. However, in the current state, the respective data have not been published in its entirety [35]. The dysfunction and pathologies of both coronary micro- and macrovasculature affect long-term graft function or dysfunction. In addition to our previous publication, the current results show that HOP with crystalloid solutions affects both the coronary micro- and macrovasculature.

### 3.4. Pathways

Enriched pathways were mainly associated with oxidoreductase activity and NADH dehydrogenase activity. Further, overrepresented pathways were related to extracellular matrix structural constituents, thus potentially suggesting superior structural integrity of the vessel. Downregulated pathways regarding cellular components indicate that the cells and the LAD cell compound may not need to be reconstructed as much in the HTK-N group as in the HTK group because they might have been injured less during HOP or reperfusion. The underrepresented pathways related to mitochondrion organization, aerobic respiration, and oxidative phosphorylation in the HTK-N group reflect a lower energy dependency of LAD cells in this group compared to the HTK group [36]. During reperfusion, the cellular metabolism is altered, increasing the energy demand [37]. The apparently higher energy demand of the LAD in the HTK group might suggest a more profound cellular injury, which could explain the vascular functional differences compared to the HTK-N group. Another possible explanation could be that the HTK-N solution potentially supported cellular recovery during HOP better than the traditional HTK solution. However, this explanation would need to be verified on a histological level.

Nevertheless, reconditioning the ischemic DCD cardiac allograft during maintenance perfusion is highly important. We showed in a previous project that HOP with HTK-N reconditions left-ventricular contractility of ischemically injured donor hearts [22]. This observed reconditioning presumes that it also happens structurally at the tissue level. Thus, the observed enrichment of pathways associated with heart development in the LAD in the present project might also be another kind of reconditioning capacity of HTK-N. However, this interpretation is limited because we did not perform a DCD group without a maintenance period.

### 3.5. Comparison of Perfusion Solutions

HTK-N was characterized by various modifications compared to the traditional HTK. Many of these modifications have been with the special purpose of protecting the coronary endothelium. The membrane-permeable iron chelator LK-614 and the membrane-unpermeable iron chelator deferoxamine were added to bind free molecular iron in a chelate complex to prevent the formation of free oxygen radicals by the Fenton reaction [38]. Adding the long-term endothelial protective amino acid L-alanine reduces oxidative stress, which is mediated by hydrogen peroxide [39]. Finally, the chloride concentration was also reduced to prevent endothelial injury mediated by chloride during reperfusion after ischemia [40].

### 3.6. Limitations

This work is limited by the fact that the microarray results were not verified at the protein level.

## 4. Materials and Methods

### 4.1. Animals and Anesthesia

The investigations were reviewed and approved (35-9185.81/G-150/19). The animals received humane care. We sedated healthy pigs (40–50 kg bodyweight) with an intramuscular injection of ketamine (22.5 mg/kg; Bremer Pharma, Warburg, Germany) and midazolam (0.375 mg/kg; Hameln pharma plus, Hameln, Germany) and maintained the anesthesia with pentobarbital sodium intravenously (15 mg/kg/h; Boehringer Ingelheim Vetmedicia, Ingelheim, Germany). For analgesia, we administered Dipidolor (1.125 mg/kg/h; Piramal Critical Care, Voorschoten, The Netherlands). We monitored the arterial blood pressure.

### 4.2. Surgical Procedure and Study Groups

**Figure 7:**
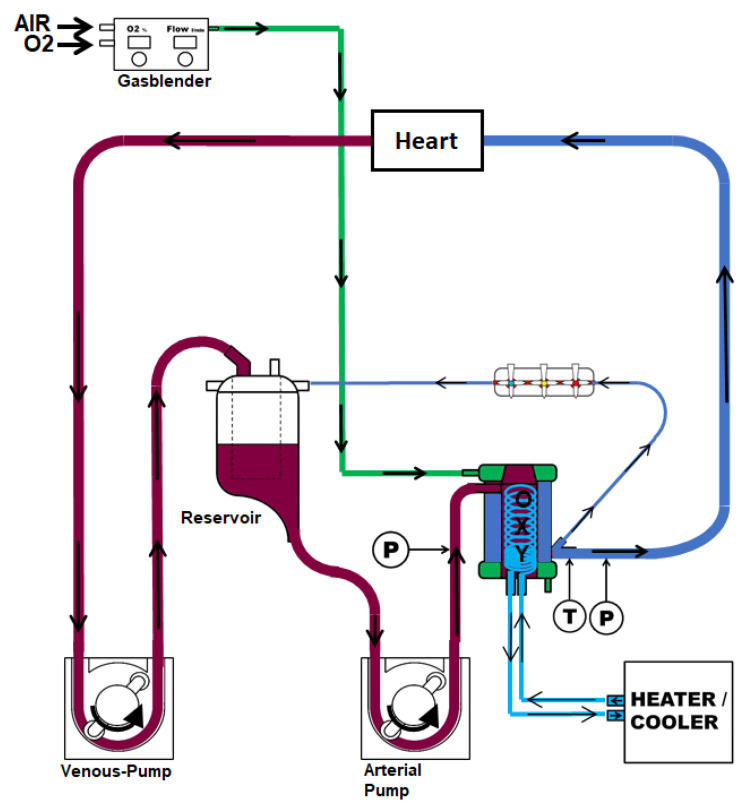
Machine perfusion system.

Femoral arterial and venous vascular access was achieved for blood sampling. Then, we performed a median sternotomy, exposed the heart, and injected heparin (LEO Pharma, Neuisenburg, Germany) intravenously. Circulatory death was induced by the termination of mechanical ventilation based on a previously published protocol [41]. We collected blood samples and harvested the heart within 30 min, starting from the termination of mechanical ventilation. After a total warm ischemic period of 30 min, we flushed the DCD hearts with 2 L of cold (4 °C) Custodiol^®^ (Köhler Chemie GmbH, Bensheim, Germany) organ preservation solution. Then, the hearts were mounted on the machine perfusion system for hypothermic, oxygenated, and crystalloid perfusion by cannulation of the ascending aorta. According to the study group, the system was either primed with traditional histidine-tryptophane-ketoglutarate (HTK, HTK-group, N = 8) or a novel HTK-N solution (HTK-N group, N = 8). The hearts were maintained by perfusion for four hours at 4 °C. The composition of the solutions is shown in the Supplementary Materials (Table S1). The hearts were immersed in the coronary effluent, which was recirculated into the perfusion system. The perfusion system comprised an open venous reservoir and a hollow fiber oxygenator (Figure 7).

### 4.3. Reperfusion and Tissue Collection

After four hours of maintenance perfusion, the hearts were reperfused with normothermic blood to mimic reperfusion after transplantation. We measured arterial and venous blood gas every 30 min (RAPID Point 500, Siemens, Erlangen, Germany). Heparin (5.000 iU), sodium chloride, magnesium chloride, glucose, sodium–prednisolone, sodium hydrogen carbonate, and mannitol were added to achieve physiological values. We adjusted the paO_2_ to 180–200 mmHg, paCO_2_ to 35–45 mmHg, and pH to 7.35–7.45. If necessary, hearts were paced at 80 beats per minute. After two hours of reperfusion, the hearts were flushed with cold Ringer solution, and the left coronary artery (LAD) was quickly excised from the left ventricle, including the surrounding myocardium, and placed on cold carbogenized Krebs–Henseleit solution (KHS). Under microscopic vision, the LAD was carefully prepared, and the surrounding myocardial tissue was removed. Finally, the LAD was dissected in four equal segments of 4 mm and mounted in organ bath chambers. Additional LAD segments were frozen in liquid nitrogen and stored at −80 °C.

### 4.4. Coronary Artery Vasomotor Analysis

The LAD rings were mounted in organ bath chambers (EMKA 4 Bath, EMKA Technologies S.A.S., Paris, France). The chambers were filled with continuously carbogenized, warm (37 °C) KHS. Initially, we equilibrated the rings for 20 min, followed by increasing the tension of the vessel periodically to 3.0 g over 60 min. During this adjustment, repeated washing steps with fresh KHS were performed. As an initial functional evaluation, we exposed the vessel rings to 80 mM potassium chloride (KCl) to test the maximal receptor-independent contractility. When the LAD rings reached a stable plateau, we washed the organ bath chambers with fresh KHS and readjusted the tension to 3.0 g. As a next functional assessment, vasoconstriction was induced with a single dose of the thromboxane A_2_ agonist U46619 (5 × 10^−9^). When the maximal contraction was reached, we used gradually increasing bradykinin (BK) concentrations to test the endothelial-dependent vasorelaxation in two rings and sodium nitroprusside (SNP) concentrations to test the endothelial-independent relaxation in two other rings.

### 4.5. RNA Preparation

We isolated total RNA from the LAD of every pig (N = 8 per group; N = 16 in total) by TRIzol (Thermo Fisher Scientific, Waltham, MA, USA;) extraction. First, we homogenized the samples using a Tissue Lyser II (Quiagen, Hilden, Germany). Then, we added chloroform to induce a phase separation. After centrifugation, we agitated the upper phase by incubation with isopropanol. Then, the RNA was pelleted by centrifugation at 4 °C. We then washed the pellet with NaAc. Then, the pellet was dissolved overnight at −20 °C in DEPC-H_2_O, followed by two washing steps with 80% ethanol for precipitation. We stored the RNA in DEPC-H_2_O at −80 °C.

### 4.6. Microarrays

We determined the LAD transcriptome using porcine arrays (Thermo Fisher Scientific, Waltham, MA, USA). First, we assessed the RNA integrity using the Bioanalyzer (2100 Bioanalyzer, Agilent, Santa Clara, CA, USA). Then, the RNA concentration was determined using a Nanodrop One (Thermofisher, Waltham, MA, USA). Biotin-labeled ss-cDNA was synthesized from total RNA with a GeneChip™ WT Pico Reagent Kit (Thermo Fisher Scientific, Waltham, MA, USA), fragmented, and subsequently hybridized using porcine arrays (Thermo Fisher Scientific, Waltham, MA, USA). Then, we washed the chips. The chips were scanned by the Affymetrix GeneChip Scanner 7G (Thermofisher, Waltham, MA, USA). One control group sample was identified as an outlier based on PCA and was, therefore, excluded. Due to the high reliability of the microarray results based on the high number of N = 8 samples per group that were individually analyzed, we did not confirm selective gene expressions by qPCR.

### 4.7. Statistical Analysis

We performed the statistical analyses using IBM SPSS Statistics for Windows (version 20.0, IBM Corp., Armonk, NY, USA). Data were presented as mean ± standard error (SEM). The nonlinear curve fit for the analysis of the dose-dependent vasoreactivity was performed using Graph Pad Prism (Version 9, Graph Pad Software Inc., San Diego, CA, USA). We used a two-tailed unpaired classical t-test in variance homogeneity and a Welch t-test in variance inhomogeneity to compare the groups. *p* < 0.05 was considered statistically significant, and a value of *p* < 0.001 was considered statistically highly significant. The Transcriptome Analysis Console (TAC 4.0; applied biosystems; Thermo Fisher Scientific, Waltham, MA, USA) was used to build heat maps and volcano plots. Differentially expressed genes were displayed through fold change (upregulated > 2.0; down-regulated < −2.0), together with a *p*-value < 0.05 using eBayes statistics. We also performed a network analysis of the top 20 downregulated genes of HTK-N vs. HTK. The network was built utilizing the Gene String online tool (STRING: functional protein association networks (string-db.org; assessed date: 18 January 2024) [42] followed by a functional annotation analyses for the identification of enriched pathways within the network [43].

## 5. Conclusions

The type of crystalloid solution used for HOP significantly affects the coronary endothelium of DCD hearts. Compared to traditional HTK, the novel HTK-N solution preserves the coronary arterial endothelial function more effectively. From this, we conclude that HOP with the novel HTK-N solution leads to improved myocardial contractility, microvascular circulation, and vasomotor function of the LAD [7,22]. The transcriptome of the LAD reveals that multiple effects seem to be the underlying reason for better endothelial function, such as decreased oxidative stress, an ameliorated I/R injury, and regenerative and mitochondria-related mechanisms. Endothelial injury remains long-term. Thus, the possible impact of HTK-N and HTK for HOP of donor hearts, especially DCD hearts, on long-term graft complications, such as CAV or graft failure, should be investigated.

## Figures and Tables

**Table 1:** Top 20 upregulated genes of HTK-N vs. HTK. Uncharacterized and pseudogenes were excluded. HTK: histidine-tryptophane-ketoglutarate. HTK-N: histidine-tryptophane-ketoglutarat-N.

Fold Change	*p*-Value	FDR *p*-Value	Gene Symbol	Description
10.36	1.78 × 10^−5^	0.0024	CEBPD	CCAAT/enhancer binding protein (C/EBP), delta
7	2.72 × 10^−5^	0.0029	CCL2	chemokine (C-C motif) ligand 2
6.89	4.92 × 10^−6^	0.0011	IER3	immediate early response 3
6.18	0.0003	0.0113	ZFP36L2	ZFP36 ring finger protein-like 2
5.87	2.68 × 10^−5^	0.0029	ACTB; ACTG1	actin, beta; actin gamma 1
5.31	0.0003	0.0114	FOXC2	forkhead box C2
5.25	1.56 × 10^−5^	0.0023	ZFP36	zinc finger protein 36, C3H type, homolog (mouse); ZFP36 ring finger protein
4.73	0.0002	0.0081	ATN1	atrophin 1
4.36	0.0001	0.0075	TMSB4X	thymosin beta 4, X-linked
4.24	9.23 × 10^−6^	0.0016	GJA1	gap junction protein, alpha 1, 43kDa
4.05	1.71 × 10^−6^	0.0008	SNAI2	snail homolog 2 (Drosophila); snail family zinc finger 2
4.02	1.46 × 10^−5^	0.0022	PNRC1	proline-rich nuclear receptor coactivator 1
3.98	7.96 × 10^−5^	0.0055	SMAD6	SMAD family member 6
3.86	0.0011	0.0258	RNF149	ring finger protein 149
3.84	0.0008	0.0211	BTG2	BTG family, member 2
3.62	0.0011	0.0262	PIM1	Pim-1 proto-oncogene, serine/threonine kinase
3.61	0.0017	0.0344	COL3A1	collagen, type III, alpha 1
3.56	0.0066	0.0783	MIR505	microRNA mir-505
3.53	0.0080	0.0874	RGS5	regulator of G-protein signaling 5
3.47	5.39 × 10^−5^	0.0044	SLC39A14	solute carrier family 39 (zinc transporter), member 14

**Table 2:** Top 20 downregulated genes of HTK-N vs. HTK. Uncharacterized and pseudogenes were excluded. HTK: histidine-tryptophane-ketoglutarate. HTK-N: histidine-tryptophane-ketoglutarat-N.

Fold Change	*p*-Value	FDR *p*-Value	Gene Symbol	Description
−39.77	6.06 × 10^−6^	0.0013	NDUFA1	NADH dehydrogenase (ubiquinone) 1 alpha subcomplex, 1, 7.5 kDa
−30.89	2.24 × 10^−6^	0.0008	TOMM7	translocase of outer mitochondrial membrane 7 homolog (yeast)
−29.51	1.93 × 10^−5^	0.0025	NDUFB2	NADH dehydrogenase (ubiquinone) 1 beta subcomplex, 2, 8 kDa
−25.59	2.87 × 10^−6^	0.0009	NDUFB1	NADH dehydrogenase (ubiquinone) 1 beta subcomplex, 1, 7 kDa
−25.48	4.18 × 10^−7^	0.0005	LOC100626068; IGIP	IgA-inducing protein
−24.94	2.62 × 10^−6^	0.0008	NDUFS4	NADH dehydrogenase (ubiquinone) Fe-S protein 4, 18 kDa (NADH-coenzyme Q reductase)
−24.47	1.98 × 10^−7^	0.0004	TBCA	tubulin folding cofactor A
−18.96	3.04 × 10^−5^	0.0032	NDUFC2	NADH dehydrogenase (ubiquinone) 1, subcomplex unknown, 2, 14.5 kDa
−18.39	2.29 × 10^−6^	0.0008	COX7A2	COX7A2 protein
−18.36	1.20 × 10^−6^	0.0007	USMG5	upregulated during skeletal muscle growth 5 homolog (mouse)
−17.63	6.49 × 10^−7^	0.0005	MGST2	microsomal glutathione S-transferase 2
−17.12	4.49 × 10^−7^	0.0005	CHCHD7	coiled-coil-helix-coiled-coil-helix domain containing 7
−16.26	3.19 × 10^−6^	0.0009	HSPE1	heat shock 10 kDa protein 1
−15.06	2.84 × 10^−7^	0.0004	PCYT1A	phosphate cytidylyltransferase 1, choline, alpha
−14.3	1.14 × 10^−5^	0.0019	NDUFB8	NADH dehydrogenase (ubiquinone) 1 beta subcomplex, 8, 19 kDa
−14.11	6.51 × 10^−6^	0.0013	ROMO1	reactive oxygen species modulator 1
−13.77	6.97 × 10^−6^	0.0014	RPL31; LOC100520127	ribosomal protein L31; 60S ribosomal protein L31
−13.42	2.89 × 10^−6^	0.0009	SNRPG	small nuclear ribonucleoprotein polypeptide G
−11.99	7.81 × 10^−5^	0.0054	RNF113A	ring finger protein 113A
−11.94	3.75 × 10^−5^	0.0035	MRPS18C	mitochondrial ribosomal protein S18C

## Data Availability

Data will be provided on reasonable request.

## Supplementary Materials

The following supporting information can be downloaded at: https://www.mdpi.com/article/10.3390/ijms25042262/s1.
