# Supplementary material for: HTK vs. HTK-N for Coronary Endothelial Protection during Hypothermic, Oxygenated Perfusion of Hearts Donated after Circulatory Death
Source: Int J Mol Sci. 2024 Feb 13;25(4):2262. doi: 10.3390/ijms25042262 (PMC10889240; doi:10.3390/ijms25042262)
Supplement: Supplementary file 1 [file ijms-25-02262-s001.zip › ijms-2813414-supplementary.pdf]

Supplementary Table S1. Composition of the maintenance solutions. HTK: histidine-tryptophane-ketoglutarate. HTK-N: Histidine-tryptophane-ketoglutarat-N.

	HTK	HTK-N
**Electrolytes**		
Na^+	16	16
K^+	10	10
Mg^{2+}	4	8
Ca^{2+}	0.015	0.020
Cl^-	50	30
**Buffer substances**		
Histidine	198	124
Nα-acetyl-L-histidine	-	57
**Further amino acids**		
Tryptophan	2	2
a-Ketoglutarate	1	2
Aspartate	-	5
Arginine	-	3
Alanine	-	5
Glycine	-	10
**Oncotic agents**		
Mannitol	30.0	-
**Sugars**		
Sucrose	-	33
**Iron chelators**		
Deferoxamine	-	0.025
LK-614	-	0.0075

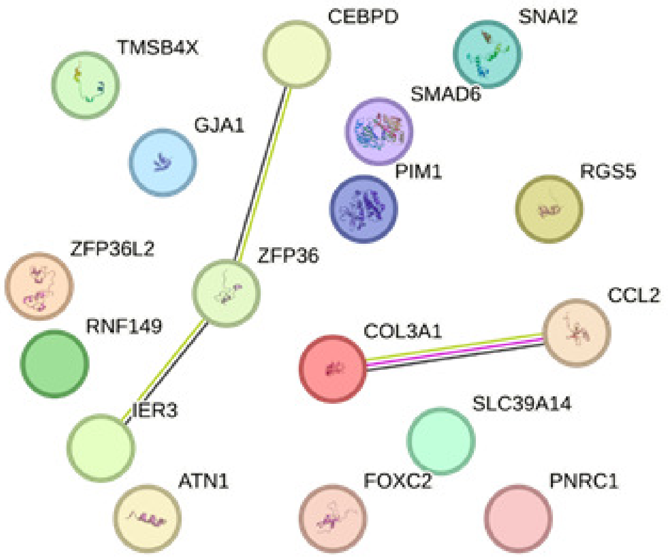

Supplementary Figure S1. Network analysis of the top 20 upregulated genes of HTK-N vs. HTK. The network was built utilizing the Gene String online tool. HTK: Histidine-tryptophane-ketoglutarate. HTK-N: Histidine-tryptophane-ketoglutarate-N.
